# Exploring the enigma within: a retrospective study of primary cardiac sarcomas from a tertiary care centre

**DOI:** 10.3332/ecancer.2025.1995

**Published:** 2025-09-24

**Authors:** Swasthik Upadhya, Sameer Rastogi, Adarsh Barwad, Shamim Ahmed Shamim, Dikhra Khan, Sudheer Arava, Vineeta Ojha, Akshit Kumar, Ganesan Karthikeyan, Akshya Kumar Bisoi

**Affiliations:** 1 Department of Medical Oncology, Dr. B. R. Ambedkar Institute Rotary Cancer Hospital, All India Institute of Medical Sciences, New Delhi 110029, India; 2 Department of Pathology, All India Institute of Medical Sciences, New Delhi 110029, India; 3 Department of Nuclear Medicine, All India Institute of Medical Sciences, New Delhi 110029, India; 4 Department of Cardiac Radiology, All India Institute of Medical Sciences, New Delhi 110029, India; 5 Department of Cardiology, All India Institute of Medical Sciences, New Delhi 110029, India; 6 Department of Cardiothoracic & Vascular Surgery, All India Institute of Medical Sciences, New Delhi 110029, India

**Keywords:** cardiac sarcoma, angiosarcoma, systemic therapy, surgical excision

## Abstract

**Background:**

Primary cardiac sarcomas are exceedingly rare tumours associated with a poor prognosis due to delayed diagnosis, advanced presentation and limited known chemotherapy efficacy. While surgical excision is the preferred treatment, it is often not feasible. The role of systemic therapy remains uncertain.

**Methods:**

We analysed the medical records of patients diagnosed with primary cardiac sarcoma registered at a sarcoma clinic between January 2016 and July 2023. Clinicopathological and imaging data and treatment information were collected. Descriptive statistics were employed for clinicopathological characteristics, and Kaplan–Meier analysis was used for survival assessment.

**Results:**

A total of 12 patients were identified with primary cardiac sarcoma, with a median age at diagnosis of 33 years (IQR 20.5; range: 17–53). At presentation, 66.7% had pericardial effusion requiring pericardiocentesis with or without pleuropericardial window. Half (6 of 12 patients) were misdiagnosed initially as either tubercular pericardial effusion or cardiac hydatid cyst. Upfront resection was done for 4 patients (33.3%), while 4 (33.3%) had locally advanced/unresectable disease and the remaining 4 (33.3%) presented with de-novo metastatic disease. Angiosarcoma constituted 50% (6 out of 12), all arising from the right atrium. Of the 12 patients included, 6 received a median two lines of therapy. Of the total 9 response assessments (both as first line and subsequent lines), 55.5% had an objective response rate with an 88.8% clinical benefit rate. The median progression-free survival for first-line systemic therapy was 5.4 months. The median overall survival for patients who received systemic therapy and the entire cohort were 19.2 and 5.1 months, respectively.

**Conclusion:**

This study highlights the advanced presentation and poor outcomes in patients with cardiac sarcoma. Due to the rarity of the tumour, it is often misdiagnosed. Systemic chemotherapy could alleviate symptoms and prolong survival. However, sarcoma pathology is heterogenous and cannot be generalised.

**Trial registration:**

As this is a retrospective observational study, no trial registration has been done.

## Introduction

Primary cardiac tumours are indeed rare conditions of the heart. In comparison, metastasis to the heart is over 20 times more common [1]. One of the largest autopsy studies, reported by Silvestri *et al* [2], which included over 1,900 patients with a primary tumour, showed cardiac metastasis in about 8% of them. Approximately 25% of primary cardiac tumours are malignant, with cardiac sarcomas comprising the majority of these malignant cardiac tumours [3]. Although cardiac sarcomas are extremely rare, almost all types of sarcomas have been documented in the literature. The most frequently described sarcomas of the heart are angiosarcoma, rhabdomyosarcoma, fibrosarcoma/undifferentiated sarcoma (formerly categorised as malignant fibrous histiocytoma or high-grade pleomorphic sarcoma) and leiomyosarcoma. Liposarcoma and synovial sarcoma have also been reported, though they are less common [4–6].

Primary cardiac sarcomas are associated with a poor prognosis due to delayed diagnosis, advanced presentation, surgical challenges, frequent metastasis and limited efficacy of chemotherapy. According to Siontis *et al* [7], approximately 46% of patients with primary cardiac sarcomas presented with metastases upfront. Because the symptoms and signs often mimic those of other benign cardiac conditions, diagnosing a cardiac tumour at an earlier stage is challenging. Many cases of cardiac sarcoma are initially misdiagnosed as benign cardiac conditions, leading to delays in timely intervention. Ouarrak *et al* [8] reported a case of cardiac sarcoma that was initially misdiagnosed as tuberculoma, resulting in the patient's death due to delayed treatment.

The clinical presentation of cardiac sarcoma primarily depends on the tumour's location in the heart rather than its histological type. Left heart sarcomas are commonly found in the left atrium and typically present with symptoms such as shortness of breath and exertional dyspnea, which result from the obstruction of pulmonary venous blood flow. As described by Rice and Reardon [9], 22 out of 24 patients (92%) with left heart sarcomas had tumours situated in the left atrium, with over 50% of patients presenting with NYHA grades III or IV dyspnea consistent with congestive cardiac failure. In contrast, right heart sarcomas tend to manifest with nonspecific symptoms and typically present with large pericardial effusion, sometimes accompanied by tamponade. As reported by Look-Hong *et al* [10], 56% of patients with right heart sarcomas presented with pericardial effusion. Embolic events are documented in approximately 25% of patients with primary cardiac tumours. Left atrial tumours, aortic valve tumours and smaller tumour burden are considered significant factors contributing to the occurrence of embolism [11]. Angiosarcoma is the most common sarcoma on the right side, whereas pleomorphic sarcoma and leiomyosarcoma are more common on the left side [12].

While cases of cardiac sarcomas are reported across all age groups, the majority of affected patients are below 65 years of age [13]. Cardiac rhabdomyosarcoma is a rare entity, with most case reports in the literature involving patients over the age of 65 [14–16]. There are very few documented cases of familial cardiac angiosarcomas; Casha *et al* [17] reported one such case in which both the patient and the patient’s father had cardiac angiosarcoma with identical histological and immunohistochemical features [17].

Complete surgical resection is the preferred treatment, but it is not feasible in most instances. Excision of a cardiac tumour via sternotomy can be highly morbid, carrying risks of infection, scarring and delays in the initiation of adjuvant chemotherapy and radiation therapy. Minimally invasive resection may be a viable option [18]. The role of chemotherapy is not well established, and most published data consist of either case reports or retrospective reviews. Therefore, the treatment of cardiac sarcomas is primarily guided by case reports and reviews. Although cardiac transplantation and cardiac auto transplantation are promising strategies, they are still in experimental realms [19].

In the present study, we aim to describe the clinicopathological and imaging findings, as well as the administered treatment and clinical outcomes of primary cardiac angiosarcoma at our institution.

## Materials and methods

This is a retrospective study in which case records of patients diagnosed with primary cardiac sarcoma registered at the sarcoma clinic between January 2016 and July 2023 were analysed. The histopathology of the cases was reported and/or reviewed by expert pathologists at our institution. After obtaining clearance from the Institutional Ethical Committee, patient data were evaluated through hospital records. Informed consent was obtained from the patients for the publication of clinical data, including histopathology images, cardiac magnetic resonance imaging (MRI) and positron emission tomography-computed tomography (PET-CT) scans. Clinicopathological characteristics and treatment details were documented. Statistical analysis was performed using SPSS 25 (SPSS, IL, USA). Descriptive statistics were used to analyse clinicopathological characteristics and the Kaplan–Meier method was utilised for survival analysis.

## Results

**Figure 1.**
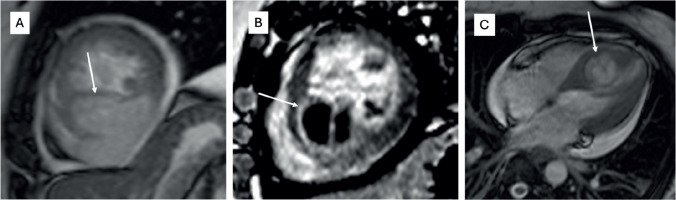
Cardiac MRI of a patient with synovial sarcoma of interventricular septum – (a): Bright blood (SSFP sequence) in short-axis view showing the heterogeneously hyperintense mass lesion (arrow) involving the septum and extending into the inferior wall of the left ventricle; (b): Late gadolinium enhancement (LGE) sequence in short axis view showing heterogenous peripheral enhancement with central non-enhancing component; (c): Bright blood (SSFP sequence) in four chamber view showing the mass (arrow) with cystic appearance within it.

**Figure 2.**
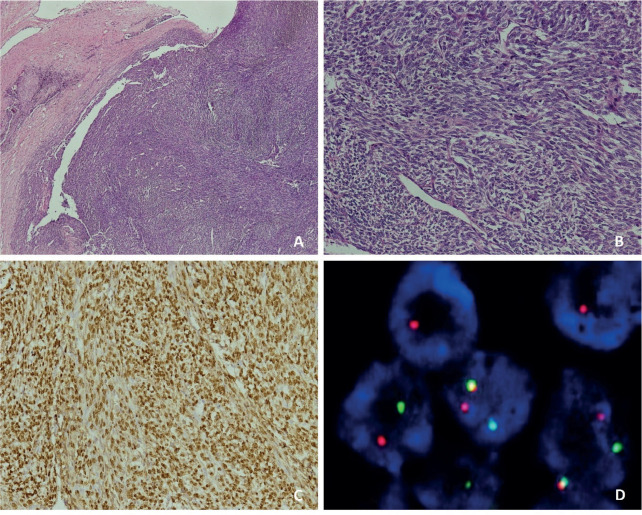
Histopathology of a 33-year-old female with cardiac synovial sarcoma synovial sarcoma – (a): Histological photomicrograph showing a spindle cell tumour with fascicular pattern of arrangement. (H&E 40×); (b): High power showing cells with spindled morphology with high N/C ratio with coarse chromatic and scanty cytoplasm. Mitosis can be noted. (H&E 200×); (c): IHC for TLE1 showing nuclear immunoreactivity; (d): FISH performed using break-part probes for SS18 gene showing spilt signals indicating SS18 gene rearrangement.

**Figure 3.**
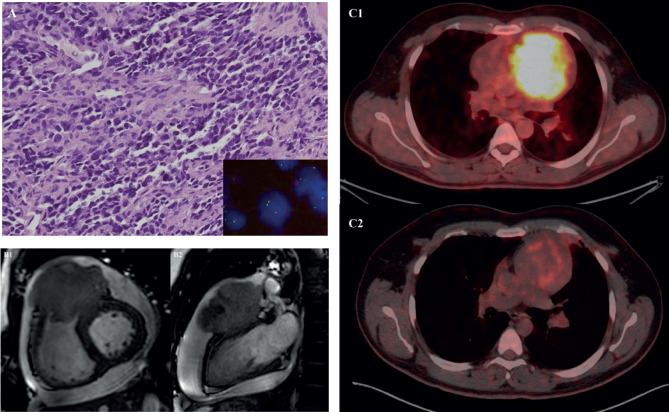
31-year-old male with desmoplastic small round cell tumour arising from right ventricular outflow tract – (a): Histological photomicrograph showing a malignant round cell tumour with undifferentiated tumour cells with high NC ratio and scanty cytoplasm. (H&E 200×); FISH performed using fusion probes for Ewsr1-WT1 gene showing fused signals indicating EWSR1-WT1 fusion (Inset); (b): Cardiac MRI – short axis view (B1) and 2 chamber view (B2) showing expansile proliferative mass lesion involving the right ventricular outflow tract; (c): FDG PET-CT before (C1) and after (C2) 6 cycles of VAC chemotherapy.

The median age of the patients was 33 years, with ages ranging from 17 to 53 years. Clinicopathological details are highlighted in Table 1. Out of the 12 patients, 7 were male (58.3%) and 5 were female (41.7%). The mean duration of symptoms was 3 months (SD 3.77). The most common symptom reported was shortness of breath (58.3%), followed by chest pain (50%). Other significant symptoms included swelling of the feet in 2 cases and facial puffiness in 1 case. Evidence of pericardial effusion was present in 7 out of 12 patients (58.3%) at presentation, and 2 patients (16.7%) had features of cardiac tamponade. Treatment details are mentioned in Table 2. The majority of patients (7 cases; 58.3%) underwent pericardiocentesis with or without a pericardial window at presentation. Only 5 patients (41.7%) underwent upfront surgical resection. Unfortunately, 6 out of 12 patients (50%) were initially misdiagnosed with a benign infective etiology at presentation (5 patients as tubercular pericardial effusion and 1 patient as a hydatid cyst (Figure 1)) with a median delay of 1.75 months (ranging from 1 to 9 months), and half of them had metastatic disease by the time the sarcoma diagnosis was made. The most common type of cardiac sarcoma in the present study was angiosarcoma, which was seen in 6 patients (50%), followed by leiomyosarcoma, synovial sarcoma (Figures 2 and 1), desmoplastic small round cell tumour (Figure 3a), hemangioendothelioma, undifferentiated pleomorphic sarcoma and low-grade fibromyxoid tumour (1 case each). The most common site of the primary tumour was the right atrium, observed in 7 out of 12 patients (58.3%) and 6 out of them were of angiosarcoma histology. Leiomyosarcoma, synovial sarcoma and desmoplastic small round cell tumour arose from the left atrium, interventricular septum and right ventricular outflow tract, respectively. About 33.3% of patients had metastatic disease at presentation, with the most common site of distant metastasis being the lung (3 out of 4 cases; 75%), while 1 patient had brain and vertebral metastasis at presentation.

Among the 4 patients who underwent upfront resection, only 1 patient achieved R0 resection and received adjuvant chemotherapy followed by radiation (Intensity modulated radiotherapy – 50.4 Gy in 28 fractions). Unfortunately, this patient succumbed due to pineal metastasis 15 months after the initial diagnosis. The other 3 patients had *R*^2^ resections, and they passed away within 2 months of surgery (2 due to metastatic disease and 1 due to perioperative complications).

For those with unresectable or metastatic disease at the time of diagnosis, palliative chemotherapy was administered. Among the 8 patients in this category, 5 (62.5%) received at least one line of palliative chemotherapy, while the remaining 3 patients (37.5%) did not receive any cytotoxic agents due to their poor performance status. The institutional practice is to administer chemotherapy based on the histology of the corresponding systemic counterpart. The most commonly used chemotherapeutic agents were single-agent Gemcitabine and single-agent Nab-paclitaxel. The median number of lines of therapy administered was 2, ranging from 1 to 3 lines. Additionally, 2 out of the 6 patients with angiosarcoma received propranolol as an additional treatment. Individual patient details are mentioned in Table 3.

**Figure 4.**
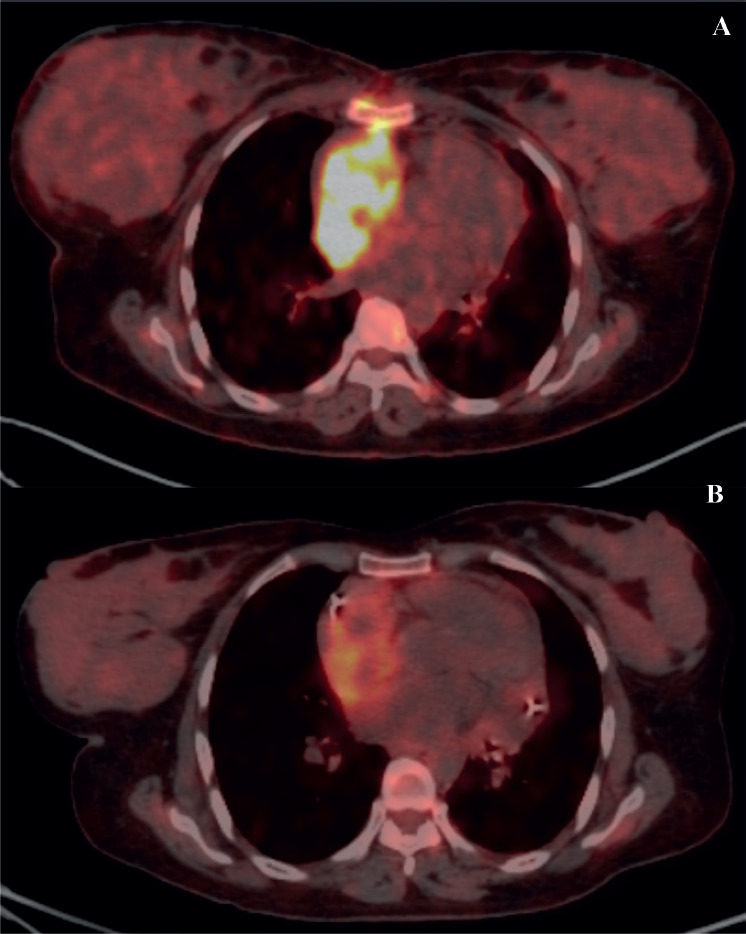
PET scans of a patient with locally advanced/unresectable cardiac angiosarcoma (a): Before chemotherapy and (b): After 5 cycles of single-agent gemcitabine.

**Figure 5.**
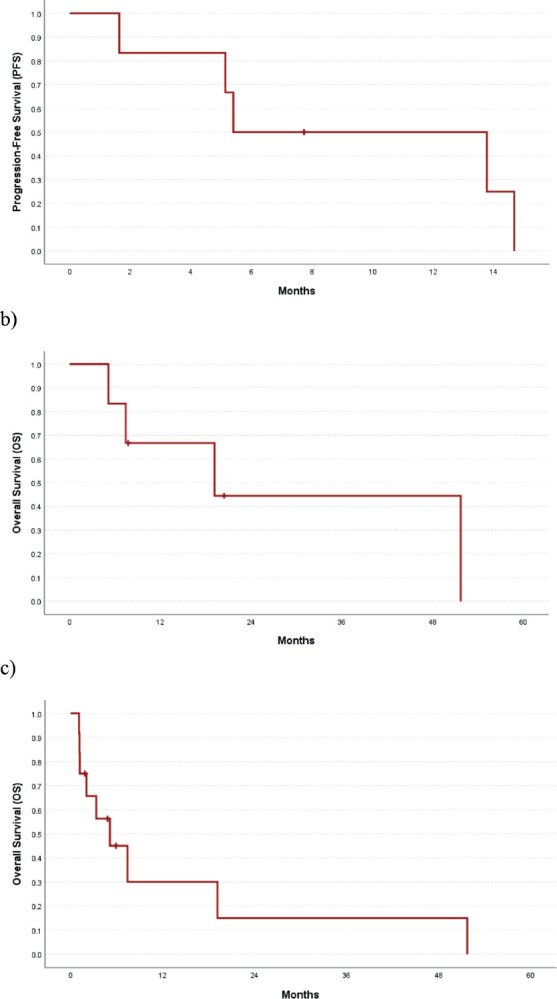
(a): PFS among those who received systemic therapy, (b): OS among those who received systemic therapy and (c): OS in the entire cohort.

Of 9 evaluable responses (both as first line and subsequent lines) among palliative setting 5 (55.5%) were PR (Figures 3c and 4), 3 (33.3%) were SD and 1 (11.2%) progression. Median progression-free survival (PFS) with systemic therapy was 5.4 months (95% CI: 0–13.7) (Figure 5a). Median overall survival (OS) was 1.2 months (those who did not receive systemic therapy) versus 19.2 months (those who received systemic therapy) (95% CI: 0–42.2) (Figure 5b). The median OS for the entire cohort was 5.1 months (95% CI: 0.2–10.0) (Figure 5c).

## Discussion

Primary cardiac sarcomas are rare and the prognosis is not encouraging. It is a disease often seen in middle-aged patients with a slight male predominance. In our study, the median age was 33 years (range 17–53 years) and had male predominance. The most common site of primary lesion in a cardiac sarcoma is right atrium and most often angiosarcoma is found to be the cause, which is reflected in the present study as well. Siontis *et al* [7] stated that 46% patients present with metastatic disease upfront. In the present case series, 33.3% had a metastatic disease at presentation. Although literature shows that some cardiac sarcomas can initially be misdiagnosed as a benign cardiac pathology, the exact percentage is not clear. In our study, 50% of patients were misdiagnosed at presentation, which led to a median delay of 1.75 months losing precious time in treatment initiation and were found to have extensive disease/metastatic disease at the time of diagnosis. This highlights that cardiac sarcomas can have certain atypical features on imaging modalities like echocardiography or cardiac MRI and hence, definitive diagnosis should always be based on histopathology. In a developing country like ours, tubercular involvement of right heart is not very uncommon and hence right-sided angiosarcomas may be misdiagnosed as the same. Also, cystic appearance of sarcoma can sometimes defy the usual diagnostic features, like in one of our cases, where it masqueraded as a hydatid cyst (Figure 1) [20].

Complete resection is the treatment of choice; however, it is not feasible in most cases. In our current series, only 4 out of 12 patients (33.3%) could undergo upfront surgical resection, of which only 1 (8.3%) had R0 resection. Median survival is typically about 6 months without surgical resection as reported by Butany and Yu [21]. However, those who undergo complete resection tend to survive for longer duration but overall prognosis remains poor. In a series of 95 patients with primary cardiac malignant tumours who underwent surgical resection, only 2 patients lived beyond 5 years [22]. There are reports of OS ranging from 12 to 30 months in patients who undergo combinations of various treatment modalities such as surgery, chemotherapy, radiation and/or transplantation [23]. In our study, median OS was 19.2 months among those who received systemic therapy. Patients with metastases at presentation have a poorer prognosis with a median OS of just over 5 months, as described by Siontis *et al* [7].

The role of chemotherapy in cardiac sarcomas is not very well established. Neoadjuvant and adjuvant chemotherapy have been used in order to improve on the poor results; however, most published experiences are either case reports or retrospective reviews. Preferred chemotherapy drugs are anthracyclines, taxanes and ifosfamide [6, 24]. In our study, 4 out of 7 (57.1%) of the patients received either single-agent Gemcitabine or Nab-paclitaxel or a combination of ifosfamide with doxorubicin. In a study of 21 patients with cardiac sarcoma, post-operative chemotherapy did not have a survival benefit in those who had incomplete resection [25]. Kakizaki *et al* [26] reported a case of a 32-year-old male with cardiac angiosarcoma who responded to multidisciplinary treatment with recombinant interleukin-2, postoperative cyclophosphamide, vincristine, doxorubicin, Dacarbazine and radiation, and at 30 months post-surgery, there was no evidence of recurrence or metastasis in spite of incomplete resection. One of our patients had a good response to multimodality treatment. Post complete surgical excision, he received adjuvant Ifosfamide with Doxorubicin and adjuvant radiation therapy. However, he succumbed to the disease relapse as pineal gland metastasis after 15 months of starting initial therapy. Since specific clinical trials concerning cardiac sarcomas are lacking, chemotherapy protocols are, in general, derived from the extracardiac soft tissue counterpart. A multinational retrospective review of 61 patients across 6 institutions in 3 continents by Chen *et al* [19] has shown an overall response rate of 32% to first-line chemotherapy [19]. On the other hand, our study has shown a 55.5% overall response rate with a clinical benefit rate of 88.8% to systemic therapy, which is exceptionally good. Weekly paclitaxel has shown excellent response in angiosarcomas with an overall tumour control rate of 70%. Nakamura-Horigome *et al* [27] have reported a case of cardiac angiosarcoma of the right atrium complicated by cardiac tamponade who responded well to combination therapy with Docetaxel and radiation with PFS of >12 months. However, in the current study, among those who received systemic therapy, the median PFS was just 5.4 months.

## Conclusion

This retrospective observational study stands as one of the largest from the Indian subcontinent, a significant achievement given the rarity of primary cardiac sarcomas. It is worth noting that there are no randomised controlled trials in the field of cardiac sarcomas, making real-world data like ours invaluable. The study highlights the advanced presentation and poor outcomes in cardiac sarcoma. Early diagnosis is of paramount importance and any typical or atypical features on imaging should be flagged so that histopathological diagnosis is not delayed. Systemic chemotherapy could alleviate symptoms and prolong survival. However, our study underscores the pressing issue of the absence of uniform criteria for treatment selection in primary cardiac sarcomas. Additionally, it prompts us to consider the potential role of chemotherapy in managing these patients, shedding light on critical areas of research and clinical practice that require further exploration and development.

## Conflicts of interest

The authors declare no conflicts of interest to disclose.

## Funding

No funding has been received for the study.

## Author contributions

SU and SR were major contributors in study conception, analysis and interpretation of results and writing the manuscript. AB and SA performed histopathological examination. SAS and DK helped in assessing the radiological responses. AKB performed the surgeries. VO and AK interpreted cardiac MRI images. VO and GK assisted in writing the manuscript. All authors read and approved the final manuscript.

## Figures and Tables

**Table 1.** Clinicopathological characteristics of primary cardiac sarcomas.

Characteristic	Value (*N* = 12)
Age	
Median	33 years
Range	17–53 years
Sex	
Male	7 (58.3%)
Female	5 (41.7%)
Presenting symptoms or signs	
Shortness of breath	7 (58.3%)
Chest pain	6 (50%)
Swelling of feet	2 (16.7%)
Facial puffiness	1 (8.3%)
Initial presentation	
Pericardial effusion Mild Moderate Severe (cardiac tamponade)	7 (58.3%) 2 (16.7%) 2 (16.7%) 3 (25.0%)
Congestive cardiac failure	2 (16.7%)
Cardiac tamponade	2 (16.7%)
Site of primary lesion	
Right atrium	7 (58.3%)
Pericardium	2 (16.7%)
Left atrium	1 (8.3%)
Interventricular septum	1 (8.3%)
RVOT	1 (8.3%)
Mean (±SD) size of the tumour in cm Antero-posterior Lateral	6.9 ± 3.8 5.3 ± 2.9
Initial diagnosis	
Tubercular pericarditis	5 (41.7%)
Cardiac tumour	6 (50%)
Hydatid cyst	1 (8.3%)
Metastasis at presentation	
No	8 (66.7%)
Yes	4 (33.3%)
Site of metastasis (*n* = 4)	
Lungs	3
Brain and vertebral	1
ECOG performance score at presentation	
0–2	6 (50%)
≥3	6 (50%)
Histopathology	
Angiosarcoma	6 (50%)
Leiomyosarcoma	1 (8.3%)
Synovial sarcoma	1 (8.3%)
Desmoplastic small round cell tumour	1 (8.3%)
Hemangioendothelioma	1 (8.3%)
Undifferentiated pleomorphic sarcoma	1 (8.3%)
Low grade fibromyxoid tumour	1 (8.3%)

**Table 2.** Treatment received.

Treatment	Value (*N* = 12)
Initial intervention at presentation	
Pericardiocentesis with/without pericardial window	7 (58.3%)
Upfront surgical resection	4 (33.3%)
Systemic therapy	
Received	6 (50%)
Adjuvant	1 (8.3%)
Palliative	5 (41.7%)
Not received	6 (50%)
Systemic therapy used (*N* = 6)	
Single agent Gemcitabine	3 (50%)
Single agent Nab-paclitaxel	2 (33.3%)
Ifosfamide + Adriamycin	2 (33.3%)
VAC	1 (8.3%)
Pazopanib	1 (8.3%)
Response to systemic therapy (among 9 responses in palliative setting)	
PR	5 (55.5%)
SD	2 (33.3%)
PD	1 (11.2%)
Other treatment received (*N* = 6)	
Propranolol	2 (33.3%)
Radiation	1 (8.3%)

**Table 3.** Details of the patients in the study.

S. No.	Age (years)/Sex	Primary site	Histopathology	FNCLCC GRADE	Clinical presentation	First diagnosis	Stage	Metastatic sites	Surgical resection	First line therapy	Best response/duration of therapy (months)	Second line therapy	Best response/duration of therapy (months)	Third line therapy	Best response/duration of therapy (months)	Current status
1.	23/F	Right atrium	Angiosarcoma	3	SOB, Massive pericardial effusion	Tubercular pericarditis	NM/unresectable	NA	No	Pazopanib	PR/12	Nab-Paclitaxel (weekly)	SD/12	Ifos-Doxo (3 cycles)	PR/22	Expired
2.	31/M	RVOT	DSCRT	3	Pericardial effusion	Cardiac sarcoma	M	Lungs	No	VAC (6 cycles)	PR/5	NA	NA	NA	NA	Expired
3.	19/M	Left atrium	Leiomyosarcoma	2	SOB	Cardiac sarcoma	NM	NA	R0 resection	Ifos-Doxo (5 cycles adjuvant) + RT (50.4 Gy/28#/5.5 weeks)	CR/12	NA	NA	NA	NA	Expired
4.	42/F	Right atrium	Angiosarcoma	3	SOB, fatigue, weight loss, pericardial effusion	Tubercular pericarditis	M	Lungs	No	Nab-Paclitaxel (weekly)	PD/0	Gemcitabine (6 cycles)	PR/4	NA	NA	Expired
5.	48/F	Right atrium	Angiosarcoma	3	SOB, facial congestion, pericardial effusion	Tubercular pericarditis	NM/Unresectable	NA	No	Gemcitabine (5 cycles)	SD/6					No follow up
6.	33/F	Interventricular septum	Synovial sarcoma	3	Chest pain	Hydatid cyst	M	Brain, vertebrae	No	NA	NA	NA	NA	NA	NA	Expired
7.	17/M	Right atrium	Angiosarcoma	3	SOB, chest pain, pericardial effusion	Tubercular pericarditis	NM	NA	*R*^2^ resection	NA	NA	NA	NA	NA	NA	Expired
8.	33/M	Right atrium	Angiosarcoma	2	Recurrent pericardial effusion	Tubercular pericarditis	NM/Unresectable	NA	No	NA	NA	NA	NA	NA	NA	Expired
9.	38/F	Right atrium	Low grade fibromyxoid/ myofibroblastic tumour	1	Dependent edema	Cardiac sarcoma	NM	NA	*R*^2^ resection	Observation	NA	NA	NA	NA	NA	Alive
10.	45/M	Pericardium	Undifferentiated pleomorphic sarcoma	3	Chest pain	Cardiac sarcoma	NM	NA	*R*^2^ resection	NA	NA	NA	NA	NA	NA	Expired
11.	53/M	Pericardium	Angiosarcoma	3	Chest pain, cardiac tamponade	Cardiac sarcoma	NM	NA	NA	NA	NA	NA	NA	NA	NA	Expired
12	23/M	Right atrium	Angiosarcoma	3	SOB, chest pain, palpitations, pericardial effusion	Cardiac sarcoma	NM/Unresectable	NA	NA	Gemcitabine (6 cycles)	PR/NA	NA	NA	NA	NA	Alive
